# IL-9 Inhibits Viral Replication in Coxsackievirus B3-Induced Myocarditis

**DOI:** 10.3389/fimmu.2016.00409

**Published:** 2016-10-06

**Authors:** Miao Yu, Qi Long, Huan-Huan Li, Wei Liang, Yu-Hua Liao, Jing Yuan, Xiang Cheng

**Affiliations:** ^1^Laboratory of Cardiovascular Immunology, Institute of Cardiology, Union Hospital, Tongji Medical College, Huazhong University of Science and Technology, Wuhan, China

**Keywords:** IL-9, viral myocarditis, coxsackievirus B3, TGF-β, coxsackie and adenovirus receptor

## Abstract

Myocardial injuries in viral myocarditis (VMC) are caused by viral infection and related autoimmune disorders. Recent studies suggest that IL-9 mediated both antimicrobial immune and autoimmune responses in addition to allergic diseases. However, the role of IL-9 in viral infection and VMC remains controversial and uncertain. In this study, we infected Balb/c mice with Coxsackievirus B3 (CVB3), and found that IL-9 was enriched in the blood and hearts of VMC mice on days 5 and 7 after virus infection. Most of IL-9 was secreted by CD8^+^ T cells on day 5 and CD4^+^ T cells on day 7 in the myocardium. Further, IL-9 knockout exacerbated cardiac damage following CVB3 infection, along with a sharp increase in viral replication and IL-17a expression, as well as a decrease in TGF-β. In contrast, the repletion of IL-9 in Balb/c mice with CVB infection induced the opposite effect. Studies *in vitro* further revealed that IL-9 directly inhibited viral replication in cardiomyocytes by reducing coxsackie and adenovirus receptor expression, which might be associated with upregulation of TGF-β autocrine effect in these cells. However, IL-9 had no direct effect on apoptosis in cardiomyocytes. Our data indicated that IL-9 played a protective role in disease progression by inhibiting CVB3 replication in the early stages of VMC.

## Introduction

Viral myocarditis (VMC) is a triphasic disease including an initial viral infection, followed by autoimmune response and finally myocardial remodeling (1). Coxsackievirus B3 (CVB3) is the common pathogen causing this inflammatory disease (2). Although excessive activation of immune response triggered by virus infection is the main factor contributing to myocardial injuries, the virus itself is critical to the progression of VMC *via* direct attack on cardiomyocytes (3).

IL-9, a cytokine produced primarily by CD4^+^ Th9 cells, is generally reported to mediate allergic and autoimmune diseases (4). Recent studies suggest that IL-9 plays an important role in infectious diseases including *Trichuris muris* expulsion and respiratory syncytial virus clearance (5, 6). For the influence of IL-9 on VMC and CVB3 infection, only Qing et al. newly observed that IL-9-secreting Th9 cells were unchanged in CVB3-induced VMC mice (7). Nevertheless, the effect of IL-9 on VMC progression and CVB3 replication remain unknown. Therefore, in this study, we investigated the expression of IL-9, viral replication, and related inflammatory factors in VMC using IL-9 knockout (IL-9KO/IL-9^−/−^) and rIL-9 injected Balb/c mice. Concurrently, the direct effects of IL-9 on myocardial cells infected with CVB3 were also studied to elucidate the mechanism involved.

## Materials and Methods

### Mice

IL-9^−/−^ mice in a Balb/c background were generated as previously described (8) and were provided by the Laboratory of Molecular Biology, Medical Research Council, Cambridge, UK. Wild-type male Balb/c mice were purchased from the Experimental Animal Center of Hubei province (Wuhan, China). All the animals were housed under standard pathogen-free conditions at the Experimental Animal Center (Tongji Medical College of Huazhong University of Science and Technology, Wuhan, China). The animal experiments were carried out according to the guidelines for the Care and Utilization of Laboratory Animals (Huazhong University of Science and Technology, China). And this study was approved by the Institutional Animal Care and Use Committee of Tongji Medical College, Huazhong University of Science and Technology according to the regulations for the administration of affairs concerning experimental animals in Hubei province of China and the constitution of the experimental animal ethics committee in Huazhong University of Science and Technology.

### Virus and CVB3 Infection

The CVB3 (3 m strain, CCTCC GDV115) titer determined by plaque-forming unit (PFU) assay in HeLa cells was 1 × 10^7^. IL-9^−/−^ and WT BALB/c mice aged 4 weeks were infected by an intraperitoneal (i.p.) injection of 0.2 mL of RPMI-1640 (Gibco) containing approximately 10^5^ PFU of CVB3 to establish the VMC models. The virus experiments were performed according to the general requirements for laboratory biosafety (GB 19489–2008) in China.

### Interventions and Groups

IL-9KO and WT BALB/c mice were divided into four groups randomly: (1) control group (*n* = 20) containing WT BALB/c mice treated with saline (300 μL per mouse); (2) WT group (*n* = 20) comprising WT BALB/c mice treated with 200 μL CVB3 and 100 μL saline; (3) IL-9KO group (*n* = 20) including IL-9^−/−^Balb/c mice treated with 200 μL CVB3 and 100 μL saline; and (4) rIL-9 group (*n* = 20) consisting of mice treated with 200 μL CVB3 and rIL-9 (1 μg diluted in 100 μL saline per mouse, PeproTech). The intraperitoneal injections of CVB3 and saline were developed on day 0. The rIL-9 was administered intraperitoneally on days 0 and 3. All the animals in each group were euthanized on days 5 and 7. The blood and hearts were removed aseptically for further measurements.

### Flow Cytometry

Hearts of mice were minced into 1 mm^3^ sections and digested with 0.1% collagenase B (Roche Diagnostics GmbH) for 6 min four times in a 37°C water bath (9). Cell suspensions were obtained by filtering through a cell strainer (40 μm size, BD Falcon) and layered over Ficoll–Hypaque density gradient solution to separate mononuclear cells for flow cytometry. Intracardiac IL-9-producing leukocytes were measured by labeling the harvested cells with the following surface markers: PE anti-mouse CD45, FITC anti-mouse CD4, FITC anti-mouse CD49b, PE-cy7 anti-mouse CD11b, PE-cy7 anti-mouse CD8, or PE anti-mouse Gr-1 antibodies (eBioscience). After washing with PBS, these cells were stimulated with 1 μg/mL ionomycin, 20 ng/mL phorbol myristate acetate (PMA), and 2 μmol/L monensin (eBioscience) for 4 h under 5% CO_2_ at 37°C in 24-hole culture plates (Costar). After washing, fixing, and permeabilizing according to the manufacturer’s instructions, the cells were stained with APC anti-mouse IL-9 antibody or isotype control antibody. The stained cells were measured and analyzed by FACScalibur flow cytometry (BD Biosciences).

### Histopathology and Immunohistochemistry

The heart was fixed in 4% paraformaldehyde for 24 h, trimmed, and embedded routinely in paraffin. Longitudinal, 5-mm-thick sections of heart were obtained for staining with hematoxylin and eosin. The severity of impairment was assessed by the percentage of cardiac sections showing inflammation compared with the overall size of the heart sections, under a microscope eye piece grid (magnification 200×) according to the following scoring system: grade 0, none; grade 1, 25% cardiac inflammation; grade 2, 25–50%; grade 3, 50–75%; and grade 4, more than 75% (10). Two independent researchers scored the results in a blinded manner.

To further evaluate the cardiac expression of IL-9, the sections were heated in a microwave using 0.01% citrate buffer (pH = 9.0) and treated with 3% H_2_O_2_ for 10 min. After washing with PBS buffer three times and blocking with 3% bovine serum albumin (BSA) for 30 min, the sections were incubated with hamster anti-mouse IL-9 IgG (eBioscience) at 4°C overnight and washed with PBS buffer three times. After incubation with HRP-conjugated anti-hamster antibody for 45 min and washing adequately, diaminobenzidine solution was added, and the sections were counterstained by hematoxylin.

### ELISA

Serum levels of IL-9, IL-17a, TGF-β, IL-10, TNF-α, IFN-γ, IFN-α, and IFN-β were determined using sensitive mouse IL-9 (Biolegengd), TNFα/IFN-γ/TGF-β/IL-10/IL-17a (Neobioscience), IFN-α (eBioscience), and IFN-β (Pbl Assay Science) kits, according to the manufacturers’ instructions. No cross-reactivity was detected. Blood concentrations of serum cardiac troponin (cTn) T were measured using a quantitative rapid assay kit (Roche Diagnostics GmbH Elecsys, Shanghai, China) as previously described (11). All the samples were measured in triplicate.

### Plaque-Forming Assay

A portion of the heart was weighed and homogenized in PBS. After three freeze-thaw cycles and centrifugation at 2000 rpm for 10 min, the supernatant was obtained and sequentially diluted 1:10 in RPMI 1640 medium. The HeLa cell monolayers were cultured in six-well plates with the supernatant for 1 h at 37°C, 5% CO_2_. They were washed in PBS and covered with 2 mL 0.4% agar, RPMI 1640, and 10% FBS (Gibco). After 72 h of incubation, the number of plaques was counted. The viral titers were analyzed by standard plaque formation assay and expressed per organ weight (gram).

### Cardiomyocyte Culture

Neonatal cardiomyocytes were isolated as previously described (12). The ventricles obtained from 1–3 days BALB/c mice were removed rapidly into cold Hanks’ balanced salt solution (HBSS). After washing and mincing, tissues were digested in 0.05% trypsin (GIBCO) for 30 min at 4°C with rotation. The tissues were transferred into DMEM (GIBCO) containing 20% FBS (Fetal bovine serum, Gibco) to terminate the digestion. After washing with HBSS, the tissues were incubated with Liberase TH (0.1 U/mL, Roche, Germany) at 37°C for 5 min, and the dissociated cells were collected into 20% FBS DMEM. This procedure was not repeated until most of the cells were released. The isolated cells were incubated with 5% CO_2_ at 37°C for 1 h. The unattached cardiomyocytes were seeded into fibronectin-coated 12-well tissue culture plates (Costar) and subsequent experiments were performed when the cardiomyocytes formed a confluent monolayer and beat in synchrony at 72 h.

### CVB3-Infecting Cardiomyocytes

The isolated neonatal cardiomyocytes were divided into three groups: (1) control group, including neonatal cardiomyocytes treated with 50 μL PBS; (2) CVB3 group, comprising neonatal cardiomyocytes incubated with CVB3 at 5 × 10^5^ PFU in 50 μL PBS; (3) IL-9 group, containing neonatal cardiomyocytes incubated with CVB3 at 5 × 10^5^ PFU in 25 μL PBS and 500 ng/mL IL-9 diluted in 25 μL PBS; and (4) IL-9 + TGF-β monoclonal antibody (mAb) group, administrating 10 μg/mL TGF-β mAb (eBioscience, 1 mg/mL) in the IL-9 group. After 48 h, the plaque-forming assay was developed as above. The protein was extracted for Western Blot test. The supernatant was tested for TGF-β, TNF-α, IFN-α, and IFN-β, using ELISA, as mentioned above.

### Apoptosis Assay

The cardiomyocytes proliferated over the coverslips in 12-well culture plates and were incubated with CVB3 at 5 × 10^5^ PFU or CVB3 + 500 ng/mL IL-9, as described above. After 12 h, the RNA was extracted and subjected to real-time PCR for Bax/Bcl-2 analysis. The TUNEL assay was performed using an *in situ* cell death detection kit (Roche) according to the manufacturer’s protocol. The TUNEL-stained slides were washed with PBS and counterstained with α-SMA (Boster, Wuhan, China) and 4′,6-diamidino-2-phenylindole (DAPI; Beyotime, Shanghai, China). A laser confocal microscope (Olympus, Tokyo, Japan) was used to acquire the images. Nuclei, which were labeled with both TUNEL and DAPI, were considered TUNEL-positive.

### Western Blot

Total proteins of the heart tissue or cardiomyocyte were extracted with the total protein extraction kit (Pierce/Thermo Scientific, USA). The BCA protein assay kit (Pierce) was used to determine protein concentrations. Samples containing 30 μg proteins were separated on a 10% SDS-PAGE and electrotransferred onto nitrocellulose membranes. The membrane was blocked for 2 h in TBST containing 5% skim milk and incubated with primary antibodies against IL-9 receptor (IL-9R, 1:500 dilution, Abcam), coxsackie and adenovirus receptor (CAR, 1:500 dilution, Santa Cruz), phosphorylated Erk1/2 (1:500 dilution, cell signaling technology), total Erk1/2 rabbit polyclonal antibody (1:1000 dilution, cell signaling technology), and beta-actin (1:1000 dilution, cell signaling technology) at 4°C over night. After washing, the membranes were incubated with HRP-conjugated secondary antibodies (1:3000) at 37°C for 2 h. The target bands were finally developed with super ECL reagent (ThermoScientific, USA), captured by Image Lab, and semi-quantitatively analyzed with densitometric methods.

### Real-Time PCR

Total RNA of heart tissue or cardiomyocyte was extracted with TRIzol reagent (Takara Biotechnology) following the manufacturer’s protocol and the PrimeScript RT reagent kit was used to reverse transcribe the RNA into DNA (Takara Biotechnology). The primers for CAR, Bax, Bcl-2, and GAPDH are listed: CAR (Sense: GCACCCGCTAAGGTAGCTG, Antis: ATAGACCCGTCCTTGCTCTGT), Bax (Sense: TGCAGAGGATGATTGCTGAC, Antis: GATCAGCTCGGGCACTTTAG), Bcl-2 (Sense: GTACCTGAACCGGCATCTG, Antis: GCTGAGCAGGGTC TTCAGAG), and GAPDH (Sense: CACGGCAAATTCAACGGC, Antis: TGATGA CCCTTTTGGCTCCA). After an initial denaturation step at 94°C for 3 min, a three-step cycle procedure (denaturation: 94°C, 30 s; annealing: 58°C, 30 s; and extension: 72°C, 30 s) was carried out for 40 cycles. The mRNA levels of target genes were quantified using SYBR Green Master Mix (Takara Biotechnology) with CFX connect real-time system (Biorad, USA). The relative level of gene expression was normalized to the level of GAPDH transcripts.

### Statistical Analysis

Data are presented as means ± SEM. Statistical analysis was performed by one-way ANOVA using SPSS 11.0, and *P* < 0.05 was considered statistically significant.

## Results

### Increased IL-9 Expression in Myocardium of VMC Mice

**Figure 1:**
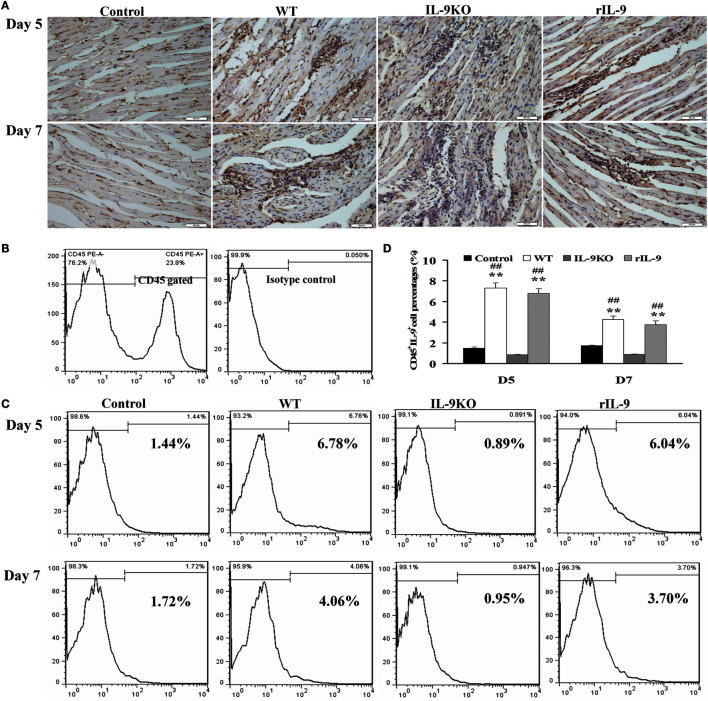

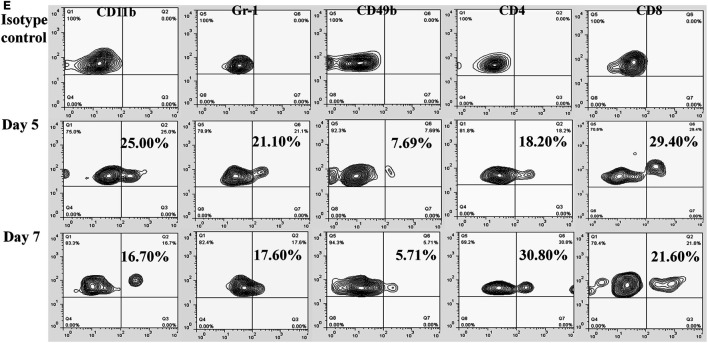
**IL-9 expressions were increased in myocardium of VMC mice**. **(A)** The results of immunohistochemistry (magnification 400×) in the heart tissue showed that IL-9 distributed in the lesions with inflammation on days 5 and 7. **(B)** CD45^+^ cells were gated. And the isotype control of IL-9 was showed. **(C)** The representative pictures for IL-9-secreting leukocyte (CD45^+^IL-9^+^) levels in different groups. **(D)** The results of statistical analysis for IL-9-secreting leukocyte levels by flow cytometry in different groups. **(E)** The CD45^+^IL-9^+^ cells were gated and further analyzed for CD11b, Gr-1, CD49b, CD4, and CD8 expressions to detect the cellular source of IL-9. **P < 0.01 vs. control group; ^##^*P* < 0.01 vs. IL-9KO group. Values are means ± SEM. Ten mice were euthanized in each group separately on days 5 and 7.

IL-9 protein expression in myocardium was enhanced on days 5 and 7 in WT and rIL-9 groups, compared with that of the control group (Figure 1A). Further, IL-9-secreting leukocyte (CD45^+^IL-9^+^) levels were significantly increased in WT and rIL-9 groups on days 5 and 7 compared with those in the control and IL-9 KO group (Figures 1B–D, all *P* < 0.01). These cells were higher in WT and rIL-9 groups on day 5 than on day 7. In addition, IL-9 protein and IL-9-secreting leukocytes in myocardium were almost not expressed in the IL-9 KO mice (Figures 1A–D). Thus, IL-9 expression was increased in myocardium of VMC mice.

To identify the leukocytes contributing to cardiac IL-9 secretion in VMC, we stained the cells with various surface markers. Most of the IL-9-secreting leukocytes were CD8-positive on day 5, and the majority of these cells were CD4-positive on day 7 (Figure 1E).

### IL-9 Attenuated the Severity of VMC

**Figure 2:**
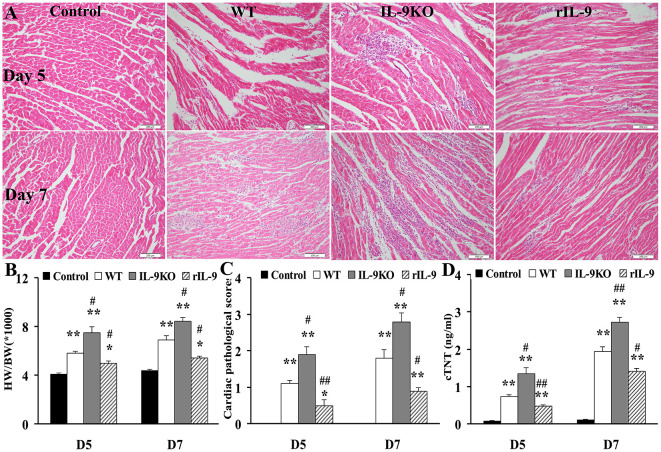
**IL-9 attenuated the severity of VMC mice**. **(A)** The representative pictures of histopathology (magnification 200×) in heart tissue. **(B)** The ratios of HM/BW in different groups. **(C)** The pathological scores in different groups. **(D)** The levels of serum cTnI in different groups. *P < 0.05 vs. control group; **P < 0.01 vs. control group; ^#^*P* < 0.05 vs. WT group; ^##^*P* < 0.01 vs. WT group. Values are means ± SEM. Ten mice were euthanized in each group separately on days 5 and 7. HM/BW, the ratios of heart weight to body weight; cTNT, cardiac troponin I.

The HW/BW (the ratios of heart weight to body weight), the pathological scores of heart sections and cTNT levels in WT, IL-9KO, and rIL-9 groups were elevated significantly compared with those in the control group (all *P* < 0.05). However, these three indices for evaluation of VMC severity were enhanced in the IL-9KO group compared with those in the WT group (all *P* < 0.05). The severity of VMC was suppressed in rIL-9 group compared with WT group (all *P* < 0.05; Figure 2). From this, we found that IL-9 could attenuate the severity of VMC.

### IL-9 Inhibited Cardiac Viral Replication in VMC

**Figure 3:**
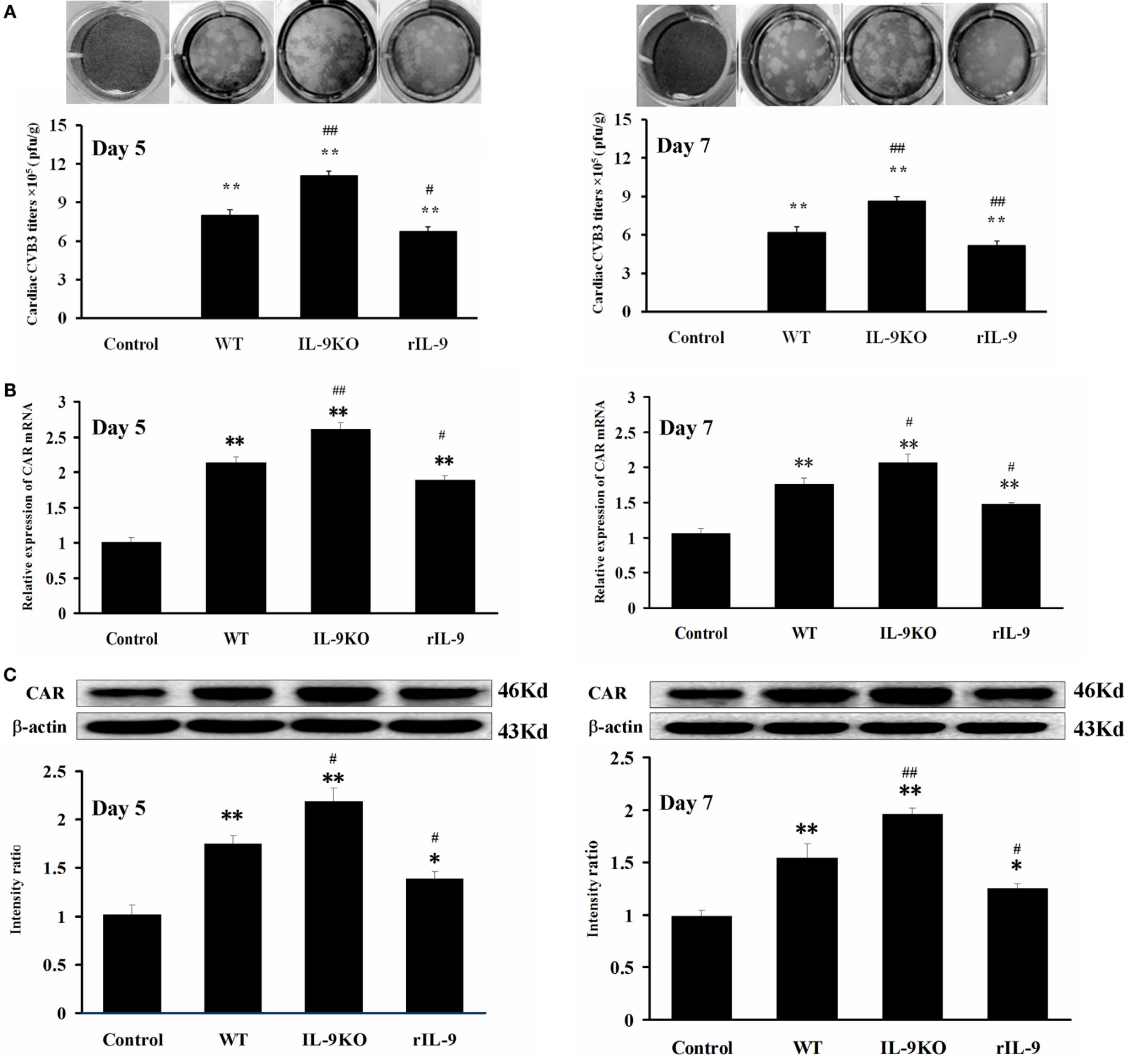
**IL-9 inhibited cardiac viral replication in VMC mice**. **(A)** The levels of cardiac CVB3 titers were showed on days 5 and 7. Data represent mean values of CVB3 PFU per gram of the hearts. **(B)** The mRNA levels of cardiac CAR expression. **(C)** The protein levels of cardiac CAR expression. *P < 0.05 vs. control group; **P < 0.01 vs. control group; ^#^*P* < 0.05 vs. WT group; ^##^*P* < 0.01 vs. WT group. Values are means ± SEM. Ten mice were euthanized in each group separately on days 5 and 7.

On days 5 and 7, the levels of cardiac CVB3 titers and CAR expressions in WT, IL-9KO, and rIL-9 groups were increased compared with those in the control group (all *P* < 0.05). The viral titers and CAR expressions in IL-9KO group were higher than those in WT group (*P* < 0.05). However, they were lower in rIL-9 group (*P* < 0.05). The control group was tested negative for cardiac CVB3 (Figure 3). These data proved that IL-9 inhibited cardiac viral replication and CAR expression in VMC mice.

### IL-9 Regulated Serum IL-17a and TGF-β Expression in VMC Mice

**Figure 4:**
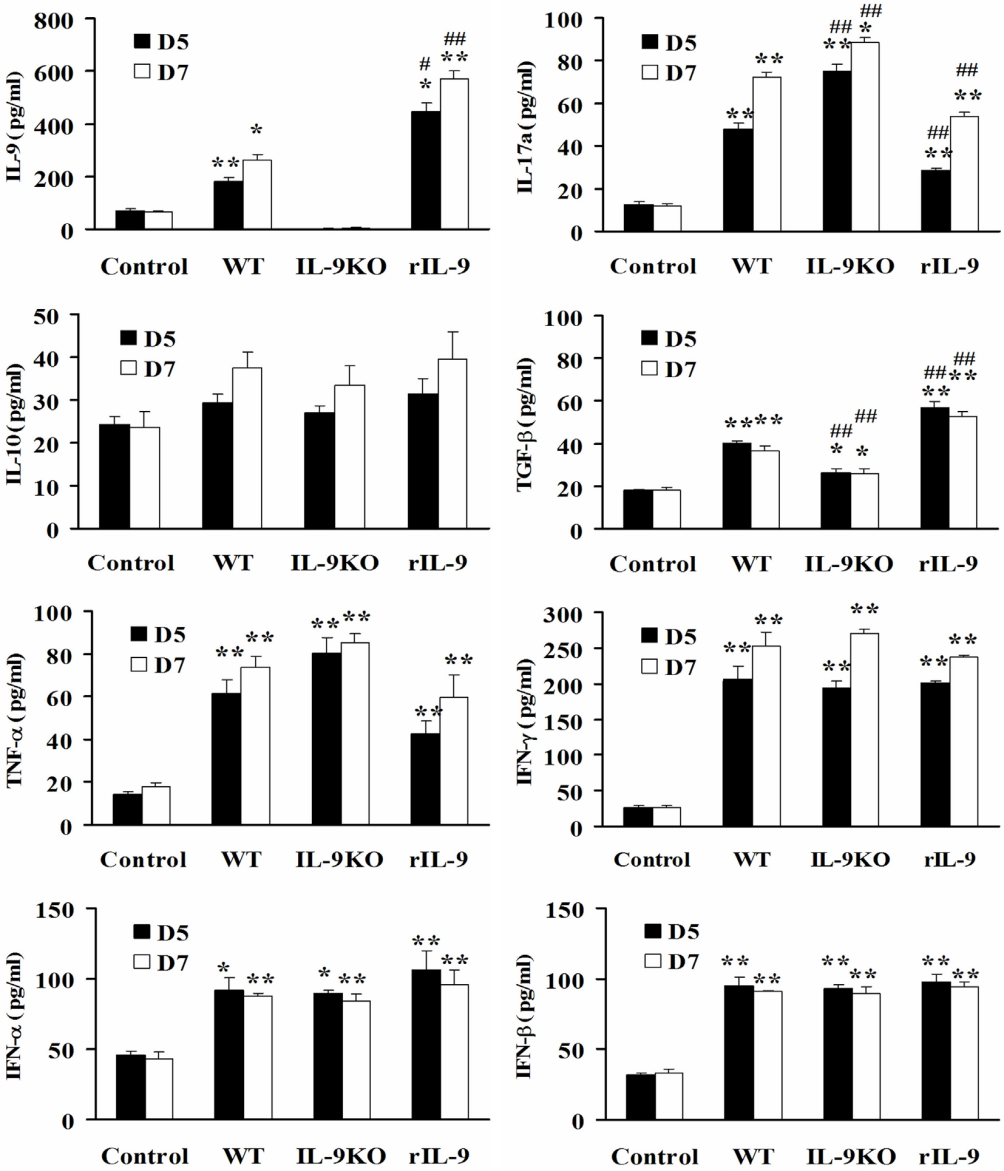
**IL-9 regulated serum IL-17a and TGF-β expressions in VMC mice**. The levels of serum IL-9, IL-17a, IL-10, TGF-β, TNF-α, IFN-γ, IFN-α, and IFN-β in Control, WT, IL-9KO, and rIL-9 groups. *P < 0.05 vs. control group; **P < 0.01 vs. control group; ^#^*P* < 0.05 vs. WT group; ^##^*P* < 0.01 vs. WT group. Values are means ± SEM. Ten mice were euthanized in each group separately on days 5 and 7.

Except for IL-10, the levels of serum IL-9, IL-17a, TGF-β, TNF-α, IFN-γ, IFN-α, and IFN-β in WT, IL-9KO, and rIL-9 groups were higher than in control mice on days 5 and 7 (all *P* < 0.05). However, the decreased IL-9 level in the IL-9KO group was accompanied by enhanced IL-17a levels and attenuated TGF-β levels compared with those in WT group (all *P* < 0.01). The opposite changes in IL-9, IL-17a, and TGF-β levels were detected in rIL-9 groups compared with those in WT group (all *P* < 0.01, Figure 4). This indicated that IL-9 downregulated IL-17a expression and upregulated TGF-β expression in VMC mice.

### IL-9 Directly Suppressed CVB3 Replication in Cardiomyocytes *In Vitro*

**Figure 5:**
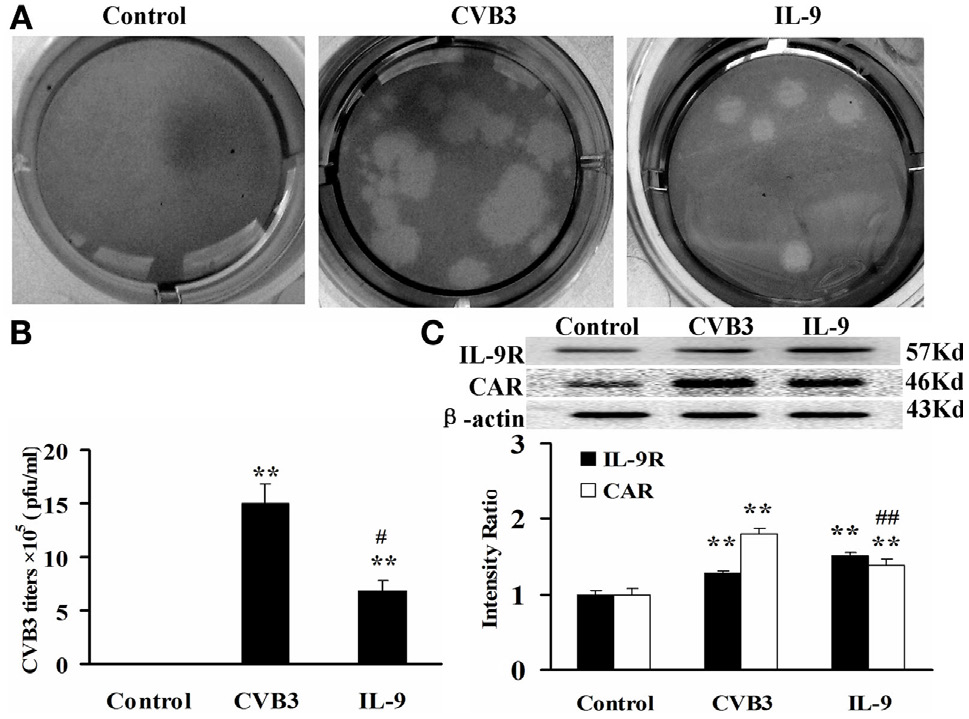
**IL-9 directly suppressed CVB3 replication in cardiomyocyte *in vitro***. **(A)** Representative pictures of plaque assay of CVB3 in different groups. **(B)** The results of statistical analysis for CVB3 titers in Control, CVB3, and IL-9 groups. **(C)** The changes of IL-9R and CAR on myocardial cells in different groups. **P < 0.01 vs. control group; ^#^*P* < 0.05 vs. CVB3 group; ^##^*P* < 0.01 vs. CVB3 group. Values are means ± SEM. Each experiment was independently performed three times.

The direct effects of IL-9 on myocardial cells were investigated *in vitro*. The neonatal cardiomyocytes were isolated, infected with CVB3, and incubated with IL-9. The CVB3 titers in the CVB3 and IL-9 groups were higher than in the control group (*P* < 0.01). However, the viral titers in IL-9 group were lower than in the CVB3 group (*P* < 0.05). CVB3 was not detected in the control group (Figures 5A,B).

To clarify the role of IL-9 in CVB3 replication in the cardiomyocytes, we determined the changes in IL-9R and CAR levels of myocardial cells. We first found that cardiomyocytes express IL-9R. Subsequently, we found that the CAR protein levels in myocardial cells were increased in the CVB3 and IL-9 groups compared with those in the control group (*P* < 0.01). They were lower in the IL-9 group than in the CVB3 group (*P* < 0.01, Figure 5C). Thus, IL-9 could directly suppress CVB3 replication and CAR expression in cardiomyocytes.

### IL-9 Facilitated TGF-β Autocrine Effect in Cardiomyocytes *In Vitro*

**Figure 6:**
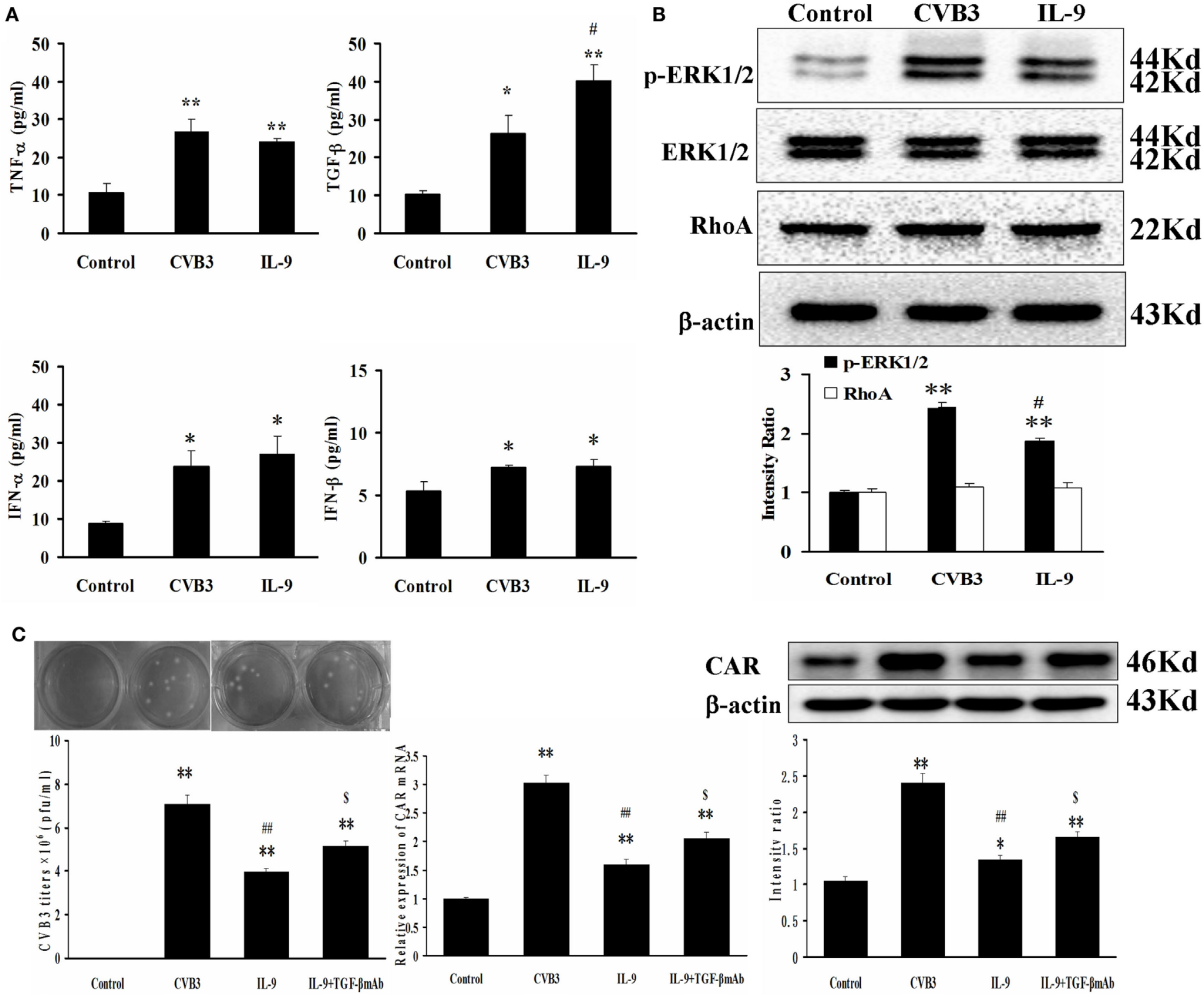
**IL-9 facilitated TGF-β autocrine effect from cardiomyocyte *in vitro***. **(A)** The levels of autocrine TGF-β, TNF-α, IFN-α, and IFN-β from myocardial cells. **(B)** The changes of signal molecule phosphorylated ERK1/2 and RhoA were showed in different groups. **(C)** The levels of CVB3 titer, CAR mRNA, and CAR protein of myocardial cells treated with IL-9 or IL-9 + TGF-β mAb. *P < 0.05 vs. control group; **P < 0.01 vs. control group; ^#^*P* < 0.05 vs. CVB3 group; ^##^*P* < 0.01 vs. CVB3 group; $*P* < 0.05 vs. IL-9 group. Values are means ± SEM. Each experiment was independently performed three times.

The autocrine effect of TGF-β, TNF-α, IFN-α, and IFN-β from myocardial cells was detected after neonatal cardiomyocytes were infected with CVB3 and incubated with IL-9. The levels of the four cytokines were higher in the CVB3 and IL-9 groups than in the control group (all *P* < 0.05). However, only TGF-β levels were higher in the IL-9 group than in the CVB3 group (*P* < 0.05). No differences in TNF-α, IFN-α, and IFN-β were found between the CVB3 and IL-9 groups (Figure 6A).

The signal molecules associated with TGF-β production in myocardial cells were investigated. The phosphorylated ERK1/2 levels were increased after CVB3 infection (*P* < 0.01) and were attenuated after IL-9 treatment (*P* < 0.05; Figure 6B). The other signal molecule RhoA was not altered following CVB3 and IL-9 interventions (Figure 6B).

Then, the TGF-β mAb in combination with IL-9 were added to further clarify the mechanisms of IL-9 on CVB3 replication and CAR expression. The data showed that the CVB3 titers and CAR expression in IL-9 + TGF-β mAb group were higher than in the IL-9 group (*P* < 0.05; Figure 6C). It further indicated that IL-9 could suppress CVB3 replication and CAR expression by facilitating TGF-β autocrine effect in cardiomyocytes.

### IL-9 Did Not Directly Influence Cardiomyocyte Apoptosis *In Vitro*

**Figure 7:**
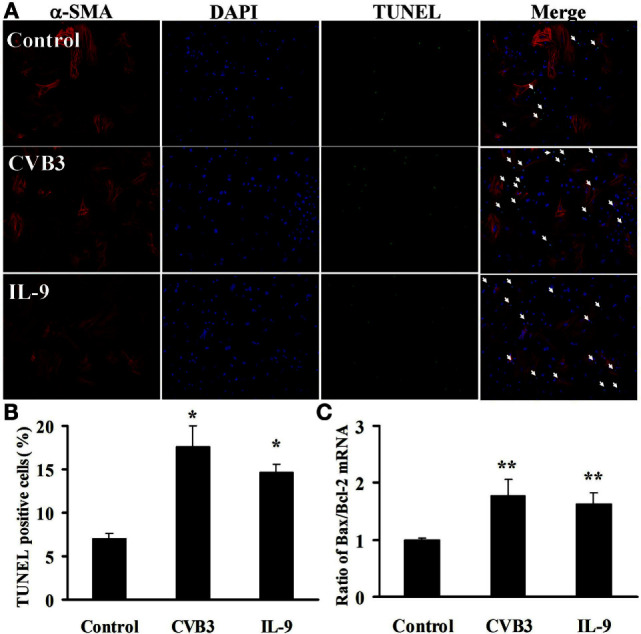
**IL-9 did not directly influence cardiomyocyte apoptosis *in vitro***. **(A)** The representative pictures for TUNEL-positive myocardial cells. α-SMA: red. DAPI: blue. TUNEL: green. **(B)** The statistical analysis for the number of TUNEL-positive cardiomyocytes. **(C)** The ratio of Bax/Bcl-2 mRNA was analyzed. *P < 0.05 vs. control group; **P < 0.01 vs. control group. Values are means ± SEM. Each experiment was independently performed three times.

To test the effects of IL-9 on cardiomyocyte apoptosis after CVB3 infection *in vitro*, we carried out TUNEL in myocardial cells. As shown in Figures 7A,B, CVB3 infection remarkably increased the number of TUNEL-positive cardiomyocytes compared with the control groups (*P* < 0.05). However, no significant differences were found in the number of TUNEL-positive cells between IL-9 and CVB3 groups.

We also examined the concurrent expression of Bcl-2 family using real-time PCR. The Bax/Bcl-2 ratio was significantly increased in cardiomyocytes exposed to CVB3 (*P* < 0.01). However, no changes in Bax/Bcl-2 ratio were found between IL-9 and CVB3 groups (Figure 7C). Thus, IL-9 did not directly influence cardiomyocyte apoptosis.

## Discussion

In this study, we first found that the serum IL-9 levels were elevated in VMC mice. Further, the number of IL-9-secreting leukocytes and IL-9 protein expression in myocardial tissue were increased on days 5 and 7 in VMC. Furthermore, most of the IL-9-secreting leukocytes were CD8^+^ on day 5 and CD4^+^ on day 7 in the myocardium, suggesting that CD8^+^ and CD4^+^ T cells might be the major source of IL-9 in VMC.

In the early stage of VMC, the direct attack by the virus was the primary cause of myocardial injury (13). We found that depletion of IL-9 facilitated virus replication along with enhanced myocardial injury. IL-9 supplementation depressed the viral replication and attenuated the myocardial injury. It suggested that IL-9 ameliorated the progression of VMC by inhibiting CVB3 replication. Cytokines play an important role in VMC by regulating antiviral immunity. IFN-γ, IFN-α, and IFN-β are the primary cytokines mediating viral death and clearance *via* macrophage activation (13, 14). TGF-β reduced viral replication and CVB3-mediated autoimmunity in the early stages of VMC (15). TNF-α is the main proinflammatory cytokine that exacerbated myocarditis through excessive autoimmunity (16). IL-17a facilitated viral replication in VMC by inhibiting IFN-γ production (17–19). The finding demonstrated that IL-9 depletion boosted IL-17a expression and inhibited TGF-β expression, while IL-9 supplementation suppressed IL-17a expression and accelerated TGF-β expression. IL-9 did not regulate the expression of IFN-γ, IFN-α, IFN-β, and TNF-α. These data indicated that IL-9 inhibited CVB3 replication by indirectly regulating IL-17a and TGF-β expression.

To further explore the direct mechanisms of IL-9 in VMC, we isolated neonatal cardiomyocytes and infected the cells with CVB3 followed by incubation with IL-9. The data suggested that IL-9R expression on cardiomyocytes and IL-9 directly inhibited CVB3 replication by binding to IL-9R. Further, administration of IL-9 reduced the levels of CAR, which is the primary receptor for CVB3 infection on cardiomyocytes. Therefore, IL-9 directly inhibited CVB3 infection by downregulating CAR expression.

The expression of CAR on cardiomyocytes was locally modulated by autocrine regulation of cytokines in myocardial cells. IFN-α, IFN-β, TNF-α, and TGF-β represent the main autocrine cytokines in cardiomyocytes (20–24). We detected changes in these cytokines and found that IL-9 only promoted TGF-β expression. The increased TGF-β level reduced CAR expression on cardiomyocytes, which suppressed CVB3 replication (16) and further suggested that IL-9 directly inhibited viral replication *via* TGF-β-CAR pathway. It is well known that the regulation of TGF-β secretion is mediated by MAPKs and Rho GTPase signaling (23). Among the MAPKs family, ERK1/2 was associated with viral replication. Luo et al. reported that CVB3 replication was reduced by inhibition of ERK1/2 signaling (25). Otsuka et al. showed that ERK1/2 inhibitors reduced TGF-β secretion in macrophages (26). However, we found that both CVB3 replication and ERK1/2 phosphorylation were decreased along with the increased TGF-β production in myocardial cells treated with IL-9. These data suggested that IL-9 induced TGF-β secretion by suppressing ERK1/2 signaling.

To further clarify the relationship among IL-9, TGF-β, CVB3 replication, and CAR expression, the TGF-β mAb was administrated in cardiomyocyte culture system. As we showed, although administration of IL-9 reduced the CVB3 replication and CAR expression along with increased TGF-β secretion, neutralization of TGF-β restored viral titers and CAR levels. These data further suggested that IL-9 could inhibit CVB3 replication and CAR expression by inducing TGF-β secretion.

Apoptosis induced by viral infection is an important mechanism limiting CVB3 replication (27). However, our study showed that IL-9 did not regulate cardiomyocyte apoptosis directly, as indicated by the change in TUNEL-positive cardiomyocytes and the ratio of pro-apoptotic (Bax) to anti-apoptotic (Bcl-2) proteins.

In this study, we found that IL-9 was locally enriched after CVB3 infection in myocardium, and depletion of IL-9 exacerbated while IL-9 supplementation ameliorated VMC. IL-9 inhibited viral replication by reducing IL-17a and enhancing TGF-β expression in VMC mice. Furthermore, IL-9 directly inhibited CVB3 replication and CAR expression by upregulating the autocrine effect of TGF-β by inhibiting ERK1/2 signaling in cardiomyocytes. We then could conclude that IL-9 play a protective role in the early stage of VMC, and IL-9 would be a novel therapeutic target for VMC. Nevertheless, the more studies were still needed for exploring the effects of IL-9 on the later stage of VMC. In addition, the investigation for roles of IL-9 in Th1 and Th17 cell differentiations might be necessary in the following study.

## Author Contributions

XC, JY, and Y-HL designed the study. MY performed the animal experiments, analyses the data, and wrote the article. QL, H-HL, and WL performed the animal and cell experiments. All authors contributed to the manuscript preparation, read, approved, and accepted the final version.

## Conflict of Interest Statement

The authors declare that the research was conducted in the absence of any commercial or financial relationships that could be construed as a potential conflict of interest.

## References

[1] Dennert R Crijns HJ Heymans S. Acute viral myocarditis. Eur Heart J (2008) 29(17):2073–82. 10.1093/eurheartj/ehn296 18617482 PMC2519249

[2] Deonarain R Cerullo D Fuse K Liu PP Fish EN. Protective role for interferon-beta in coxsackievirus B3 infection. Circulation (2004) 110(23):3540–3. 10.1161/01.cir.0000136824.73458.20 15249500

[3] Liu PP Mason JW. Advances in the understanding of myocarditis. Circulation (2001) 104(9):1076–82. 10.1161/hc3401.095198 11524405

[4] Kaplan MH Hufford MM Olson MR. The development and in vivo function of T helper 9 cells. Nat Rev Immunol (2015) 15(5):295–307. 10.1038/nri3824 25848755 PMC4445728

[5] Dodd JS Lum E Goulding J Muir R Van Snick J Openshaw PJ. IL-9 regulates pathology during primary and memory responses to respiratory syncytial virus infection. J Immunol (2009) 183(11):7006–13. 10.4049/jimmunol.0900085 19915054

[6] Richard M Grencis RK Humphreys NE Renauld JC Van Snick J. Anti-IL-9 vaccination prevents worm expulsion and blood eosinophilia in *Trichuris muris*-infected mice. Proc Natl Acad Sci U S A (2000) 97(2):767–72. 10.1073/pnas.97.2.767 10639154 PMC15405

[7] Qing K Weifeng W Fan Y Yuluan Y Yu P Yanlan H. Distinct different expression of Th17 and Th9 cells in coxsackie virus B3-induced mice viral myocarditis. Virol J (2011) 8:267. 10.1186/1743-422x-8-267 21635745 PMC3315794

[8] Townsend JM Fallon GP Matthews JD Smith P Jolin EH McKenzie NA. IL-9-deficient mice establish fundamental roles for IL-9 in pulmonary mastocytosis and goblet cell hyperplasia but not T cell development. Immunity (2000) 13(4):573–83. 10.1016/S1074-7613(00)00056-X 11070175

[9] Liao YH Xia N Zhou SF Tang TT Yan XX Lv BJ Interleukin-17A contributes to myocardial ischemia/reperfusion injury by regulating cardiomyocyte apoptosis and neutrophil infiltration. J Am Coll Cardiol (2012) 59(4):420–9. 10.1016/j.jacc.2011.10.863 22261166 PMC3262985

[10] Nishio R Matsumori A Shioi T Ishida H Sasayama S. Treatment of experimental viral myocarditis with interleukin-10. Circulation (1999) 100(10):1102–8. 10.1161/01.CIR.100.10.1102 10477536

[11] Metzler B Mair J Lercher A Schaber C Hintringer F Pachinger O Mouse model of myocardial remodelling after ischemia: role of intercellular adhesion molecule-1. Cardiovasc Res (2001) 49(2):399–407. 10.1016/S0008-6363(00)00261-3 11164850

[12] Yu M Hu J Zhu MX Zhao T Liang W Wen S Cardiac fibroblasts recruit Th17 cells infiltration into myocardium by secreting CCL20 in CVB3-induced acute viral myocarditis. Cell Physiol Biochem (2013) 32(5):1437–50. 10.1159/000356581 24296428

[13] Wang YX da Cunha V Vincelette J White K Velichko S Xu Y Antiviral and myocyte protective effects of murine interferon-beta and -{alpha}2 in coxsackievirus B3-induced myocarditis and epicarditis in Balb/c mice. Am J Physiol Heart Circ Physiol (2007) 293(1):H69–76. 10.1152/ajpheart.00154.2007 17434974

[14] Jin B Wang RY Qiu Q Sugauchi F Grandinetti T Alter HJ Induction of potent cellular immune response in mice by hepatitis C virus NS3 protein with double-stranded RNA. Immunology (2007) 122(1):15–27. 10.1111/j.1365-2567.2007.02607.x 17451465 PMC2265985

[15] Shi Y Fukuoka M Li G Liu Y Chen M Konviser M Regulatory T cells protect mice against coxsackievirus-induced myocarditis through the transforming growth factor beta-coxsackie-adenovirus receptor pathway. Circulation (2010) 121(24):2624–34. 10.1161/circulationaha.109.893248 20530002

[16] Yamada T Matsumori A Sasayama S. Therapeutic effect of anti-tumor necrosis factor-alpha antibody on the murine model of viral myocarditis induced by encephalomyocarditis virus. Circulation (1994) 89(2):846–51. 10.1161/01.CIR.89.2.846 8313574

[17] Yuan J Cao AL Yu M Lin QW Yu X Zhang JH Th17 cells facilitate the humoral immune response in patients with acute viral myocarditis. J Clin Immunol (2010) 30(2):226–34. 10.1007/s10875-009-9355-z 20012175

[18] Yuan J Yu M Lin QW Cao AL Yu X Dong JH Th17 cells contribute to viral replication in coxsackievirus B3-induced acute viral myocarditis. J Immunol (2010) 185(7):4004–10. 10.4049/jimmunol.1001718 20802148

[19] Yuan J Yu M Lin QW Cao AL Yu X Dong JH Neutralization of IL-17 inhibits the production of anti-ANT autoantibodies in CVB3-induced acute viral myocarditis. Int Immunopharmacol (2010) 10(3):272–6. 10.1016/j.intimp.2009.11.010 19932195

[20] Li L Sherry B. IFN-alpha expression and antiviral effects are subtype and cell type specific in the cardiac response to viral infection. Virology (2010) 396(1):59–68. 10.1016/j.virol.2009.10.013 19896686 PMC2787694

[21] Noah DL Blum MA Sherry B. Interferon regulatory factor 3 is required for viral induction of beta interferon in primary cardiac myocyte cultures. J Virol (1999) 73(12):10208–13. 10559337 10.1128/jvi.73.12.10208-10213.1999 PMC113074

[22] Takahashi N Calderone A Izzo NJ Jr Maki TM Marsh JD Colucci WS. Hypertrophic stimuli induce transforming growth factor-beta 1 expression in rat ventricular myocytes. J Clin Invest (1994) 94(4):1470–6. 10.1172/jci117485 7929822 PMC295284

[23] Xiao YQ Freire-de-Lima CG Schiemann WP Bratton DL Vandivier RW Henson PM. Transcriptional and translational regulation of TGF-beta production in response to apoptotic cells. J Immunol (2008) 181(5):3575–85. 10.4049/jimmunol.181.5.3575 18714031 PMC2583327

[24] Yu X Deng L Wang D Li N Chen X Cheng X Mechanism of TNF-alpha autocrine effects in hypoxic cardiomyocytes: initiated by hypoxia inducible factor 1alpha, presented by exosomes. J Mol Cell Cardiol (2012) 53(6):848–57. 10.1016/j.yjmcc.2012.10.002 23085511

[25] Luo H Yanagawa B Zhang J Luo Z Zhang M Esfandiarei M Coxsackievirus B3 replication is reduced by inhibition of the extracellular signal-regulated kinase (ERK) signaling pathway. J Virol (2002) 76(7):3365–73. 10.1128/JVI.76.7.3365-3373.2002 11884562 PMC136021

[26] Otsuka M Negishi Y Aramaki Y. Involvement of phosphatidylinositol-3-kinase and ERK pathways in the production of TGF-beta1 by macrophages treated with liposomes composed of phosphatidylserine. FEBS Lett (2007) 581(2):325–30. 10.1016/j.febslet.2006.12.032 17222412

[27] Zaragoza C Saura M Padalko EY Lopez-Rivera E Lizarbe TR Lamas S Viral protease cleavage of inhibitor of kappaBalpha triggers host cell apoptosis. Proc Natl Acad Sci U S A (2006) 103(50):19051–6. 10.1073/pnas.0606019103 17138672 PMC1748175

