# Corrigendum: IL-9 inhibits viral replication in coxsackievirus B3-induced myocarditis

**DOI:** 10.3389/fimmu.2024.1495232

**Published:** 2024-10-16

**Authors:** Miao Yu, Qi Long, Huan-Huan Li, Wei Liang, Yu-Hua Liao, Jing Yuan, Xiang Cheng

**Affiliations:** Laboratory of Cardiovascular Immunology, Institute of Cardiology, Union Hospital, Tongji Medical College, Huazhong University of Science and Technology, Wuhan, China

**Keywords:** IL-9, viral myocarditis, coxsackievirus B3, TGF-β, coxsackie and adenovirus receptor

In the published article, there was an error in **Figure 2A** as published. The representative pathological picture for D5 WT group in **Figure 2A** was incorrectly placed by mistake. The corrected **Figure 2A** and its caption appear below.

**Figure 2:**
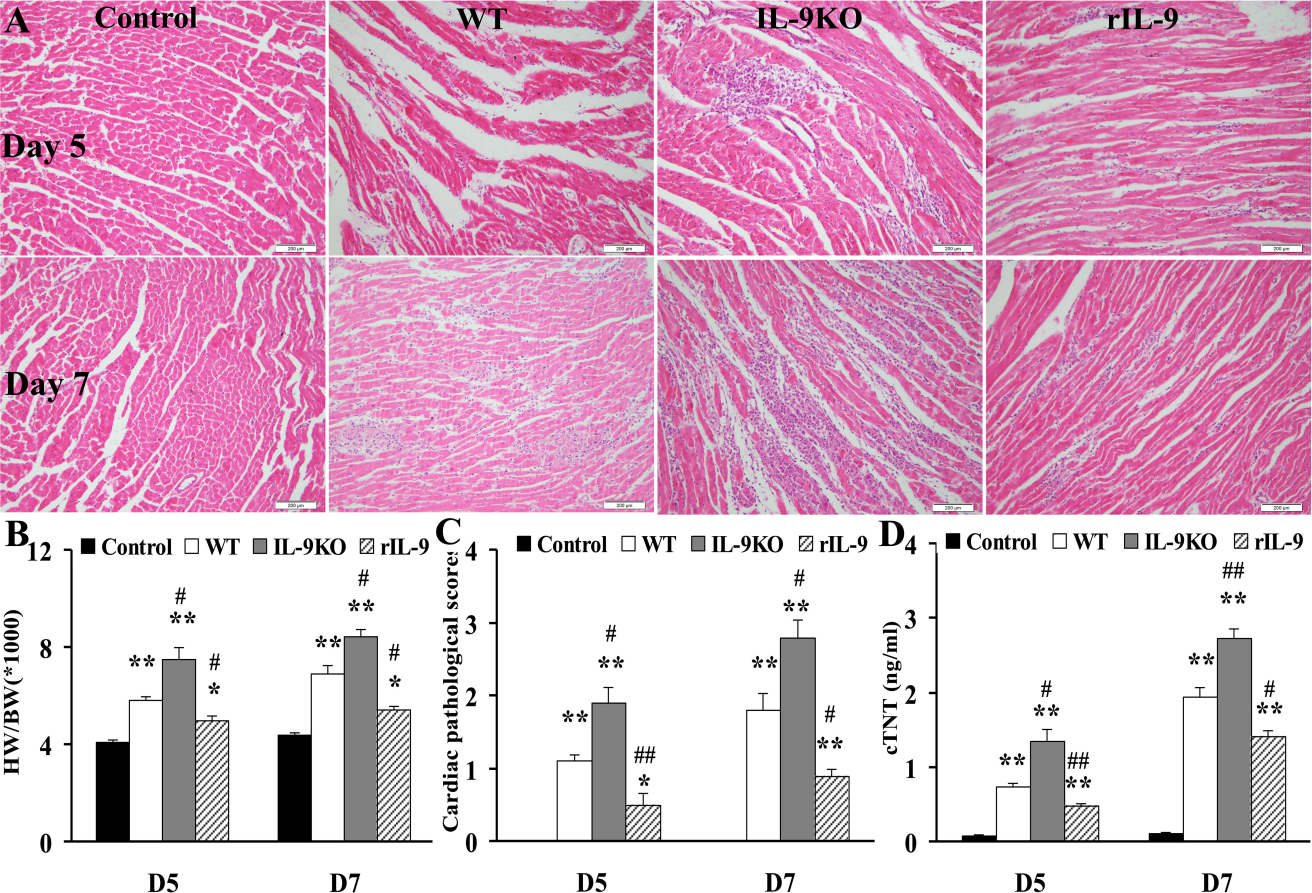
IL-9 attenuated the severity of VMC mice. **(A)** The representative pictures of histopathology (magnification ×200) in heart tissue. **(B)** The ratios of HM/BW in different groups. **(C)** The pathological scores in different groups. **(D)** The levels of serum cTNT in different groups. *P<0.05 vs. Control group; **P<0.01 vs. Control group; #P<0.05 vs. WT group; ##P<0.01 vs. WT group. Values are means ± SEM. Ten mice were euthanized in each group separately on days 5 and 7. HM/BW, the ratios of heart weight to body weight; cTNT, cardiac troponin I.

The authors apologize for this error and state that this does not change the scientific conclusions of the article in any way. The original article has been updated.

